# Impact of using routine healthcare data on the efficiency of implementation trials: a qualitative comparative case study

**DOI:** 10.1186/s13063-026-09706-3

**Published:** 2026-04-11

**Authors:** Charis Xuan Xie, Alice-Maria Toader, Anna De Simoni, Sandra Eldridge, Joseph E. Glass, Hilary Pinnock, Clare Relton

**Affiliations:** 1 Wolfson Institute of Population Health, Queen Mary University of London, London, UK; 2 MRC-NIHR Trials Methodology Research Partnership, Department of Health Data Science, University of Liverpool, Liverpool, UK; 3 Kaiser Permanente Washington Health Research Institute, Seattle, WA USA; 4 Chestnut Health Systems, Lighthouse Institute, Eugene, OR USA; 5 Centre for Population Health Sciences, Usher Institute, The University of Edinburgh, Edinburgh, UK

**Keywords:** Implementation trials, Routine healthcare data, Trial efficiency

## Abstract

**Background:**

Randomised implementation trials evaluate the effects of implementation strategies on implementation outcomes and may also monitor clinical effectiveness. Routine healthcare data are used in implementation trials for participant identification, intervention delivery, and/or outcome ascertainment. Trial efficiency (scientific, operational, statistical, and economic) is operationalised across trial design, processes, superstructure, infrastructure, and stakeholder engagement (the Trial Efficiency Pentagon). Despite frequent usage, the contribution of routine data to implementation trial efficiency remains underexplored. We aimed to investigate how the use of routine healthcare data affects trial efficiency in two implementation trials.

**Methods:**

We conducted a qualitative comparative case study of two implementation trials, one UK-based and one US-based. Participants were purposively sampled from trial teams involved in the use and management of routine healthcare data. Data were collected through semi-structured interviews, document analysis, and feedback workshops. Framework analysis guided by the Trial Efficiency Pentagon was used to analyse the data, and data flow diagrams were developed to visualise routine data pathways within each trial.

**Results:**

The two trials (DIGITS and IMP^2^ART) used routine data to characterise the practice population of eligible patients, support clinical and economic outcome evaluation, facilitate audit and feedback, and assist in intervention delivery. Common facilitators that supported the use of routine data included sufficient IT and hardware capacity, relatively low cost, centralised regulatory approval for multi-site studies, and strong collaboration and partnerships. Common barriers included administrative complexity, redundant bureaucratic processes, and challenges with data sharing requirements. Key differences included the DIGITS trial’s in-house data warehouses within an integrated healthcare system ensured high data quality and enabled preliminary analyses. In contrast, the IMP^2^ART trial, managing a larger national sample, employed an external research database to integrate data from various EHR systems but faced challenges such as legacy systems, diverse coding practices and site-specific approvals. Data quality can act as either a facilitator or a barrier.

**Conclusions:**

Routine data has an impact on implementation trial efficiency across trial design, processes, superstructure, infrastructure, and stakeholder engagement. To improve trial efficiency in public healthcare systems, researchers must address technological and regulatory barriers to accessing data. In private healthcare systems, data use and access hinges on investing in robust IT infrastructure and ensuring comprehensive organisational commitment.

**Trial registration:**

IMP^2^ART trial registration: ISRCTN15448074; DIGITS trial Clinicaltrials.gov Identifier: NCT05160233.

**Supplementary Information:**

The online version contains supplementary material available at 10.1186/s13063-026-09706-3.

## Contributions to the literature

- Provides empirical evidence on how routine healthcare data has an impact on multiple dimensions of trial efficiency within implementation trials.
- Applies the Trial Efficiency Pentagon as an analytical framework, offering researchers and practitioners a structured approach to examine and enhance trial efficiency through the five key aspects of trial design, trial process, infrastructure, superstructure, and stakeholder engagement.
- Highlights contextual facilitators and barriers, showing that challenges and enablers differ significantly between public and private healthcare systems.

## Introduction

Healthcare data such as electronic health records (EHRs), administrative datasets and patient registries are regularly collected from routine clinical practice [1]. In particular, the widespread adoption of EHRs has significantly increased the availability of routine healthcare data, making it a valuable resource for research and healthcare improvement [2]. In the UK, the National Health Service (NHS) offers universal healthcare, collecting the longitudinal medical history of approximately 98% of the population [1]. In the US, the Health Information Technology for Economic and Clinical Health Act (HITECH) of 2009 was a significant catalyst in promoting the use of EHRs, leading to a robust infrastructure for collecting and utilising routine healthcare data across various healthcare settings [2]. In both healthcare systems, routine data are promoted as essential for enhancing the efficiency of healthcare trials [3], including implementation trials [4].

Implementation trials evaluate the effects of implementation strategies on implementation outcomes and may also monitor clinical effectiveness [5, 6]. Routine healthcare data are increasingly used in implementation trials for participant identification, outcome assessment, and intervention delivery [4]. However, the successful use of these data requires careful attention to data quality, system interoperability, and research governance [4]. Although routine healthcare data are frequently expected to improve trial efficiency, existing evidence remains fragmented and conceptually heterogeneous. A key aspect is that trial efficiency is a multidimensional concept that is rarely examined in a systematic or integrated way, making it difficult to assess how routine data influence efficiency in implementation trials.

**Fig. 1:**
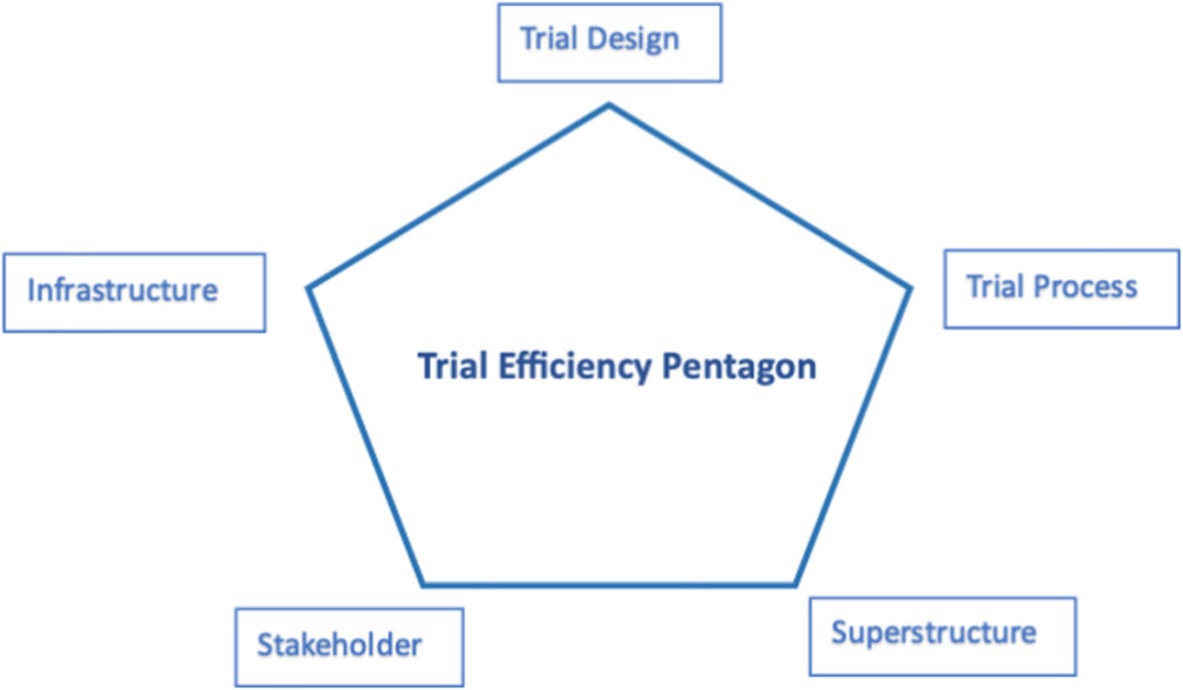
Trial efficiency pentagon

Previously we developed a conceptual framework to define trial efficiency through a Delphi study involving trial teams, funders, sponsors, journal editors, and public representatives [7]. This framework articulates trial efficiency through four theoretical constructs: scientific efficiency, operational efficiency, statistical efficiency, and economic efficiency. These constructs are operationalised through five empirical building blocks: trial design, trial process, infrastructure, superstructure, and stakeholders (known as the Trial Efficiency Pentagon) [7]. Figure 1 provides a visual overview of the framework and its five building blocks. By grounding the abstract constructs in practical aspects, the Pentagon provides a comprehensive approach to evaluating the efficiency of clinical trials.

This study aims to use the Trial Efficiency Pentagon to explore how the use of routine healthcare data affects trial efficiency in two diverse implementation trials. It seeks to answer the following questions: How are routine data operationalised within two implementation trials? What barriers and facilitators have an impact on efficiency when using routine data in these trials? What are the similarities and differences between the two trials in terms of routine data usage?

## Methods

### Study design

This study adopts a qualitative comparative case study design [8], which is well suited to addressing exploratory research questions focused on understanding how routine healthcare data are used and what factors influence trial efficiency within real-world implementation trials. Two implementation trials were examined: IMP^2^ART in the UK [9] and DIGITS in the US [10]. Comparison was used to examine how routine healthcare data are operationalised across different trial contexts and healthcare systems, with a focus on identifying patterns, facilitators, and barriers relevant to trial efficiency. The Trial Efficiency Pentagon was used as a guiding analytical framework to structure data collection and analysis across the two cases. Consistent with a case study approach [8], data were collected through semi-structured interviews, documentation analysis and feedback workshops with trial team members from February 2024 to September 2024. This study adheres to the consolidated criteria for reporting qualitative research (COREQ) guidelines [11] (Additional file 1). The ethics approval was provided by Queen Mary University of London research ethics committee (QME23.0070).

### Case selection and context

We explored how routine healthcare data affects trial efficiency in two implementation trials, one in the UK [9] and the other in the US [10] (Table 1). A ‘case’ was defined as a specific application of routine healthcare data within an implementation trial, including its use for participant identification, outcome assessment, intervention delivery, or combinations thereof. Cases were selected purposefully to capture variation in how routine data are used across different healthcare systems and data infrastructures. Both trials met predefined criteria of being randomised implementation trials that made substantive use of routine data and were either ongoing or recently completed, allowing for in-depth exploration of contemporary experiences. The contrast between a publicly funded healthcare system and a private health system provided an opportunity to examine how contextual differences shape the use of routine data and its implications for trial efficiency.

**Table 1:** Key case characteristics

	**CASE A**	**CASE B**
Trial Name	IMP^2^ART (IMPlementing IMProved Asthma self-management as RouTine)	DIGITS (Digital Therapeutics for Opioids and Other Substance Use Disorders)
Context	UK NHS	US Kaiser Permanente
Settings	Primary care practices across England and Scotland	Primary care clinics in Washington State
Trial Design	Hybrid type II cluster-randomised implementation trial	Hybrid type III cluster-randomised implementation trial
Sample Sizes	144 primary care practices	22 primary care clinics
Evidence-based Interventions	Supported asthma self-management	Digital therapeutics (reSET® and reSET-O®) for treating substance use disorders
Implementation Strategies	Professional training, patient resources, organisational support (including audit and feedback; an asthma review template)	Practice facilitation and health coaching
Outcome Measures	Implementation outcome (ownership of asthma action plans); clinical outcomes (unscheduled care, asthma control)	Reach and fidelity of digital therapeutics (reSET® and reSET-O®), treatment engagement, and population-level cost-effectiveness
Routine Data Type	EHR	EHR, healthcare insurance claims, vendor-supplied digital therapeutic data
Routine Data Usage	- Assessing clinical outcomes - Questionnaire mailing - Conducting health economic analysis - Assisting in intervention delivery (audit and feedback, EHR templates)	- Assessing clinical and implementation outcomes - Monitoring intervention delivery - Assisting in intervention delivery (audit and feedback, EHR templates)

### Sampling and recruitment

Participants were recruited from the research teams involved in the two implementation trials. Individuals were identified through discussion with the principal investigators based on their direct involvement in the use of routine data, including roles related but not limited to trial design, data management, analysis, governance, and intervention delivery. Convenience and snowball sampling was used to capture a range of perspectives across different trial roles and areas of responsibility, such as principal investigators, trial managers, statisticians, health economists, and members of the public. Potential participants were emailed information and invited to discuss the project with the lead researcher (CX), with informed consent requested via an electronic form.

### Data collection

Primary data were collected via audiotaped semi-structured interviews, by CX (female, PhD student with experience of qualitative research methods). Secondary data were collected from trial documentation to capture the historical context and understand the official narratives and discourses within different cases [12]. Documents, including trial protocols, amendments, grant applications, publications, institutional review board (IRB) and ethics applications, and publicly accessible online materials, were collected from trial managers in March 2024. These materials facilitated familiarisation with the trials and preparation for interviews, providing a foundational understanding for conducting informed discussions.

The initial interview guide was pilot-tested with five experts from various organisations, including Queen Mary University of London (QMUL), the Institute of Cancer Research, and the National Institute for Health and Care Research who held roles such as chief investigators, statisticians, and research operations officer. Interviews were conducted between February and July 2024. Topic guides provided a structure for discussions, with questions based on the five building blocks of the trial efficiency framework and refined iteratively during data collection. Each interview lasted 40 to 60 min, and was audio-recorded and transcribed verbatim. See Additional file 2 for interview topic guide.

Following the interviews and preliminary analysis, feedback workshops were conducted with participants from each trial team to present the emerging findings and to gather feedback for refinement and validation. Two online workshops were held in September 2024, one with the DIGITS trial team and one with the IMP^2^ART trial team, to allow for discussion within each trial context. Each workshop lasted approximately one hour and involved group discussion of the preliminary results.

### Data analysis

Qualitative data were collated and stored centrally for coding using ATLAS.ti (Version 24.1.1). The iterative data analysis was conducted by CX from May to September 2024. Framework analysis [13] was employed for its systematic approach to thematically organise and interpret qualitative data. Data were grouped into explanatory codes and placed under appropriate analytical themes, elucidating the barriers to, and facilitators of, using routine data in implementation trials. Following the initial coding, a framework matrix was developed to organise the data systematically, with rows representing themes and codes and columns representing each case. A random sample of 10% of the interviews was independently coded by a second coder (AMT) to check coding consistency. Any discrepancies were resolved by consensus or in consultation with the research team. In addition, we presented and discussed the preliminary findings in feedback workshops with the study participants as a form of member checking [14], to validate and refine the results.

The Trial Efficiency Pentagon was applied as an a priori analytical framework to guide data collection and framework analysis. While the framework informed coding and interpretation, the analysis was not constrained by the predefined domains, and attention was given to identifying and documenting any themes that fell outside the framework.

Due to the small sample size and to maintain participants' confidentiality, we report short or fragmented quotes and, when necessary, paraphrased quotes that were too specific while ensuring key points were retained. Participants were assigned identifiers such as P1, P2, etc., based on the order of their interviews. Since some documents are not publicly available, document codes were assigned sequentially in the order they were referenced in the analysis (D1, D2, etc.).

Simplified data flow diagrams were used to illustrate the flow of routine data in the case study implementation trials. They provide a clear and concise approach for illustrating the stakeholders involved and how data moves between them.

## Results

Ten participants (five from each trial) took part in the interviews, including principal investigators, health economists, statisticians, programmers, trial managers and lay members. These ten team members represented a selection of key personnel involved in the management of routine data use for each case. Two feedback workshops were held, the first included the DIGITS trial team, focusing on the discussion of routine data in the US context; the second included the IMP^2^ART trial team, discussing routine data in the UK context.

**Fig. 2:**
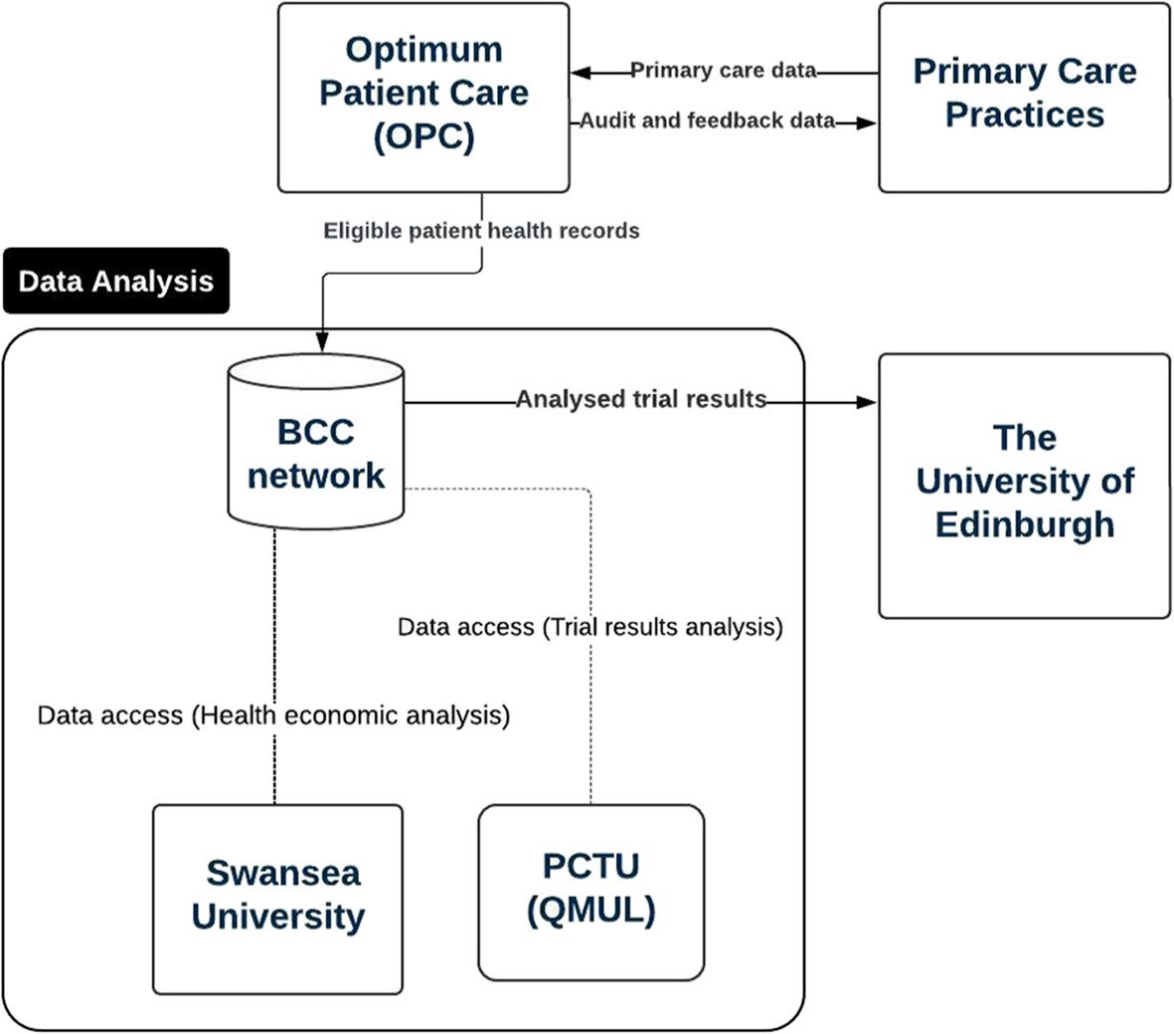
IMP^2^ART trial data flow. Figure note: The BCC network refers to the Barts Cancer Centre (BCC), which is part of Queen Mary University of London (QMUL). Specifically, it is a secure research database that manages and stores various types of clinical trial data and health-related research projects. OPC: Optimum Patient Care; PCTU: Pragmatic Clinical Trials Unit; OPC provides eligible patient health records to the BCC network and audit and feedback data to Primary Care Practices. PCTU and Swansea University access data within BCC for trial results and health economic analysis, respectively. The University of Edinburgh receives analysed trial results only

**Fig. 3:**
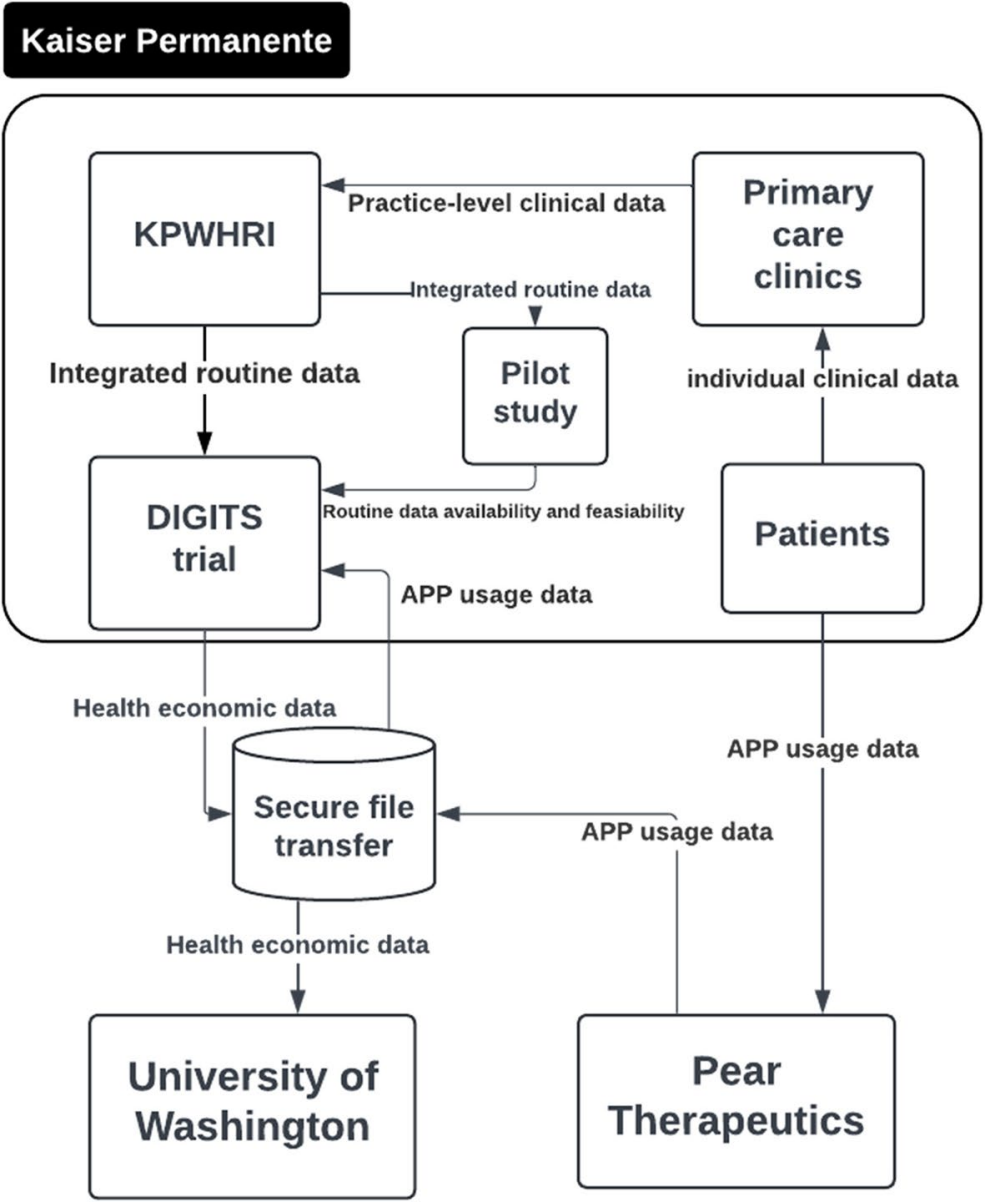
DIGITS trial data flow. Figure note: KPWHRI stands for Kaiser Permanente Washington Health Research Institute, which provides integrated routine data to support research activities; Pear Therapeutics is a digital therapeutics company providing evidence-based interventions (reSET® and reSET-O®) in DIGITS trial

Two distinct models of using routine data were identified from two trials (see Fig. 2 and Fig. 3). The primary difference lies in whether the EHR data were sourced externally or internally. In the IMP^2^ART model (Fig. 2), EHR data were extracted by an external research database managed by Optimum Patient Care (OPC; https://www.optimumpatientcare.org/) from 144 independent primary care practices using a range of clinical software. In contrast, DIGITS trial (Fig. 3) is rooted within the Kaiser Permanente (KP) infrastructure, allowing it to use EHR and healthcare claims data from the internally managed Kaiser Permanente Research Data Warehouse, and it also obtained routinely generated data the digital therapeutic vendor.

The following section presents the results from the framework analysis of qualitative data, highlighting the similarities and differences across two cases, organised according to the five building blocks of trial efficiency. To enhance readability, all relevant quotes are provided in Table 2.

**Table 2:** Exemplar quotes supporting themes and sub-themes in two cases

Themes	Sub-themes	DIGITS Quotes	IMP^2^ART Quotes
	**Facilitators**		
**Trial Design**	**Characterising practice population of eligible patients**	“Patient eligibility criteria for automatic inclusion are determined with EHR data” (D2) [10]	“Our patient population is […] all patients with a diagnosis of ‘active’ asthma. The only exclusions which we will apply in defining our patient populations from routine data are[…]” (D6) [9]
	**Preliminary analyses inform trial design**	“We did a secondary data analysis before the full trial […] this allowed us to inform our coding decisions and understand how much outcome data we could expect.” (P6)	N/A
	**Inclusion of large sample size**	N/A	“[…] to deliver this trial not using routine data — that would just not have been possible for 144 sites.” (P10)
	**Supporting outcome assessment**	“[…]without routine data I think a lot of implementation outcomes like the observable ones really can’t be effectively measured” (P6)	“we are using routine data from primary care practices for our outcomes” (P1)
	**Supporting health economic evaluation**	“Intervention costs include encounters with clinicians related to the initiation and continued use of reSET and reSET-O. We will identify patient visits with app prescribers and health coaches […] using EHR data.” (D2) [10]	“it (routine data) saved the day with the health economic analysis” (P7)
	**Barriers**		
	**Risk of identification bias**	“The way we assign patients can influence who gets identified and could potentially bias the outcomes” (P3)	N/A
	**Lack of routine data for assessing implementation outcomes**	N/A	“For outcomes not available from routine data (ownership of action plans), we will use responses to the Optimum Patient Care (OPC) quality improvement questionnaires sent to a random sample of patients …..” (D1)
**Trial Process**	**Facilitators**		
	**Timely data for audit and feedback**	“Monthly, there was the standard implementation report that tracks prescriptions and module usage across clinics, and a monthly practice facilitation report that provides detailed information to support facilitators in coaching social workers.” (P4)	“[…] for the audit and feedback we're having every month” (feedback workshop)
	**Adaptive data management**	“once the first batch of data is collected we don’t wait until the end of the data collection” (P8)	N/A
	**Standardised templates for intervention delivery**	EHR documentation templates are part of the implementation toolkit (D2, D3)	Components of the implementation strategy include educational modules, facilitator training, a range of accessible action plans, and asthma review templates (D4)
	**Barriers**		
	**Administrative complexity**	"[…] when you get into waiver requests, there are different entities that require us to meet different criteria.” (P5)	“so every single one of those applications for those 144 practices […] required a whole package of information which […] the team had to provide individually for each of those 144 practices.” (P2)
	**Lack of real-time data for interim analysis**	N/A	“We’re not doing it (data collection and analysis) in chunks” (P10)
**Infrastructure**	**Facilitators**		
	**In-house data warehouses**	"We have a virtual data warehouse, there’s also an enterprise data warehouse[…] So there’s actually a programming team that maintains a lot of the databases and programmes, variables so that they can be more efficiently used." (P6)	N/A
	**Integrated healthcare system ensures good standards of data quality**	“Something that I’ve found really useful is that the data is not in isolation, it’s in a health system.” (P3)	N/A
	**External research database integrates data from different EHR systems**	N/A	Optimum Patient Care Research Database receives pseudonymised data from practices who have agreed for the data they provide to be used for approved research purposes. OPCRD has NHS research ethics committee (REC) approval to provide access to anonymised research data for studies with scientific or patient benefits that have ethics approval. (D5)
	**IT and hardware capacity**	“we have a dedicated team with an audience of thousands of programmers and hundreds of studies over decades looking at the same common data model” (P4)	All trial data will be uploaded onto a dedicated folder on the secure virtualised environment at the Barts Cancer Centre (BCC) which is where all analysis of the PCTU trial data is carried out. (D1)
	**Cost-effectiveness**	“we had an expectation that the trial would be less costly […] because we’re part of KP we don’t pay those costs (for accessing routine data)” (P6)	“so you can get permission to go into practices and manually look up information and note it down on paper anonymously […] we worked out how much it would cost and how long it would take, and how many researchers we’d need to actually go and do manually what we're doing electronically and the answer was huge. […] (using routine data) is not cheap but it is cheaper than employing six researchers to go around and do it on a piece of paper.” (P1)
	**Data quality**	*Completeness*: “Because outcomes are ascertained from the EHR, we will have complete outcome data on all patients” (D3) *Interoperability*: “the data quality is really good, I guess because we’re an integrated system, we do have access to almost everything related to the patients, claims data, EHR data and things like that.” (P8)	*Missing data:* Missing values are likely to be extremely few as we are using routinely collected data from patients on the practice ‘active asthma’ register at all three timepoints (i.e. eligible for the supported self-management intervention throughout the trial) extracted at 24 months after randomisation. (D1) “[…] we don’t account for missing data here – if an unscheduled care event is available in this coding then we assume it’s yes – if there is no coding we assume there hasn’t been an unscheduled care event.” (P10)
	**Barriers**		
	**Legacy EHR systems**	N/A	“The incompatible systems –- legacy systems, where a practice had an old version of EHR systems […] it was not compatible for OPC to sort of set up the processes that were required to extract the data.” (P2)
	**Coding standard changes**	N/A	Over the next few years, there will be a shift from the Read codes currently used in primary care to SNOMED-CT. (D4) “we could use them in the main trial – can pre-specify them and have it all ready but the problem was that the pilot was done on read codes and not SNOMED and we need to do this transition and redefine them basically. That was something that happened that was out of our control.” (P10)
	**Data quality**	*Potential coding variation*: “I guess sometimes you get, well it depends, if you get a data that has like a variable could have, I mean an exposure or a cover could have like five different definitions, it’s just like which one do I use?” (P8)	*Availability*: “The OPC doesn’t routinely collect quality of life data like that, so we can’t use it. So again it takes it back to if you have to do that trade off” (P7) *Granularity*:” It’s (routine data) been widely used and it’s getting better. […]one of the challenges that we particularly had – is what we call the granularity of it” (P7) *Inconsistency*: “So sometimes I could go in with an asthma attack, and you could go in with an asthma attack, and we would be recorded completely, differently in terms of codes. There's no consistency codes.” (P9)
**Superstructure**	**Facilitators**		
	**Centralised regulatory approval for multi-sites study**	“To comply with the rules we’re only required to do one, so we don’t for instance have to apply to the State because we’re not using State data, we’re just using data that is accessible to the healthcare system.” (P6)	“we have moved to a centralised ethics permission (via the Integrated Research Application System)” (P2) OPC already have Research Ethics Committees (REC) approval for the use of their database for research purposes […] we had approval from the OPC’s ethics committee […] and we were advised by our University sponsor and the NHS R&D office (NHS Lothian) that we did not require any additional approval for the use of OPC data. (D4)
	**Barriers**		
	**Redundant bureaucratic processes**	"[…] a lot of it is repetitive when you get into the waiver requests because it’s for different entities that we have to meet different criteria […]" (P5)	“[…] hours and hours and hours and hours and hours went into getting all the approvals and getting it set up.” (P1)
	**Site-specific approval process from health boards or trusts**	N/A	“The team had to prepare and send individualised packages of information for 144 different practices, each with slightly different requirements. […] We had submitted 65 documents […] in a large number of separate emails to different health boards. […] But they’re all sitting there in the HRA(Health Research Authority: the national body responsible for overseeing research ethics and approvals in the UK) because we obviously submitted them.” (P2)
	**Multiple IRB applications with varying risk levels**	“We had like 10 different applications, some of them were lower risk than others […] but the actual trial did go to that full committee […] because of the fact that we had a lot of waivers of consent and we were gathering data for a lot of people, they wanted to make sure that we had the proper protections in place.” (P6)	N/A
	**Data sharing**	“There’s recent emphasis on data sharing. So there’s data sharing plans that studies need to have, that could be really challenging using routine data as it’s not the studies data. It’s the health systems data.” (P3)	"we’ve had to work out a data sharing agreement, which is another governance process to get the data from OPC to the PCTU […] when you have to work with legal contract teams then that really does slow everything down. And contracts have been back and forth between different institutions to make sure that all the legal teams are happy with what we're saying […] I think saying that we're transferring a flat CSV file from one place to another sounds very straightforward, but it actually isn’t.” (P1)
	**Practice-level data privacy concerns**		“The two practices that withdrew were concerned about where their practice-level data was going […]” (feedback workshop)
**Stakeholders**	**Facilitators**		
	**Collaboration and teamwork**	"[…] when we get data that we’re not sure about, we can work with people who are actually involved in the provision of care and figure out how to define data, and what makes sense” (P3) ”[…] there is constant communication between the team members about analytical variables” (P8)	"I'd say that there was a lot of collaboration given the amount of meetings and the amount of paperwork, and the amount of discussions, and the back and forth.“ (P9)
	**Patient and Public Involvement (PPI)**	N/A	Providing a lay perspective on validating anonymised electronic routine data by manual extraction from patient records. Her (the PPI colleague) contribution helped us balance the benefits/risks of using data from healthcare records, and ensuring commensurate safeguards. (D4)

### Trial design

Routine data enhanced the trial design for both cases. Both are cluster trial designs, and EHRs played a crucial role in defining and characterising the practice population of eligible patients, allowing researchers to understand the overall eligibility of patients within each cluster. In particular, the DIGITS trial used routine data to perform preliminary analyses that informed the trial design and ensured its feasibility. However, it faced a potential risk of identification bias, as clinics receiving the intervention might be more likely to diagnose substance use disorders. The reliance on routine data for identifying eligible patients could contribute to an overrepresentation of such diagnoses in the intervention group compared to the control group, leading to challenges in accurately defining and comparing clinic-level populations [15]. For the IMP^2^ART trial, employing EHR data allowed the inclusion of large sample size (144 practices across England and Scotland), which contributed to the robustness and power of the trial design, as well as the potential for more generalisable findings.

Routine data also facilitated the definition and collection of outcome measures, though in slightly different ways in each case. In the DIGITS trial, EHR data supported the assessment of both implementation outcomes and clinical outcomes; while the IMP^2^ART trial leveraged EHR data to evaluate clinical outcomes and conduct the health economic analysis, but was unable to assess the implementation outcomes of ownership of action plans because the coding was likely to have been influenced in the implementation group practices compared to the control practices (See Table 2 for examples).

### Trial process

In both trials, EHR data facilitated monthly audit and feedback for practice performance. The DIGITS Trial required clinicians to use referral order technology to log each prescription and request co-signature by a physician. This technology included templated text with required elements to facilitate a prescription. Both trials provided standardised templates to support intervention delivery which would ensure consistent data capture within routine clinical practice. The use of intervention delivery documentation templates was at the discretion of practices, allowing flexibility in adoption. Both trials experienced administrative complexity when requesting EHR data, involving excessive paperwork that required substantial time and resources and posed challenges to trial operations and slowed data integration into trial workflows (see Table 2 for details).

In the DIGITS trial, the collection of real-time EHR data provided a distinct advantage by enabling reporting to healthcare partners, which allowed for timely adjustments and close monitoring of the trial process. Meanwhile, adaptive data management facilitated continuous refinement of data processing throughout the trial, ensuring that the data collection remained responsive to emerging needs. In contrast, in the IMP^2^ART trial, the full dataset will be received at the end of the trial, limiting the ability to make timely adjustments and making the trial process less flexible overall.

### Infrastructure

The biggest difference between the two cases was the infrastructure. DIGITS trial benefited from an in-house routine data warehouse specifically designed for research purposes. This centralised data repository, integrated within Kaiser Permanente's healthcare system, ensured consistent and high-quality data, as well as low-cost data access. The integrated healthcare system allowed for seamless data flow and management, supporting the trial’s needs for real-time data collection and analysis.

In contrast, IMP^2^ART relied on an external research database (Optimum Patient Care Research Database). OPC extracted data from the 144 practice EHR systems, which reduced the costs associated with manual data extraction. Sufficient IT and hardware capacity within the BCC network at QMUL was available to manage the large-scale data requirements. However, the trial encountered significant infrastructure barriers. For example, legacy EHR systems in a few practices could not be extracted because they were not compatible with newly updated information governance requirements, preventing these practices from participating despite their willingness. Additionally, a recent change in coding standards within the UK EHR systems, transitioning from Read codes to Systematised Nomenclature of Medicine Clinical Terms (SNOMED CT) codes. This required complex conversion algorithms, which increased the workload and complicated data analysis.

Data quality serves as both a facilitator and a barrier for the two cases. The DIGITS trial demonstrated strengths such as the complete outcome data and enhanced interoperability due to an integrated system. However, it faced possible challenges like variations in coding definitions which could affect data interpretation (see examples in Table 2). Similarly, the IMP^2^ART trial highlighted minimal missing data at the patient level, but encountered limitations at the practice level, with a few practices withdrawing due to concerns about data extraction. Additionally, data consistency and granularity presented challenges for supporting implementation outcomes.

### Superstructure

For both trials, the centralised regulatory approval process for using routine data streamlined the initial stages of ethical and regulatory oversight to some extent. However, in the IMP^2^ART trial, this efficiency was offset by the need to secure site-specific approvals for using practice data from each of the 144 health boards and trusts where the trial was conducted. This decentralised process, along with variations in governance priorities in different regions complicated the trial. There were also data privacy concerns about the use of practice-level data, leading to practice withdrawals from the IMP^2^ART trial. As for DIGITS trial, the study required multiple IRB applications due to the different activities involved. The higher-risk applications, particularly those involving routine data with waivers of consent, faced additional scrutiny and extended the approval timeline. Data sharing emerged as another common challenge, underlined by the recent emphasis on sharing study data publicly and complex legal requirements for internal data sharing between collaborating institutions (see Table 2 for examples).

### Stakeholders

Both teams highlighted that effective stakeholder engagement was a key factor in their trial. The DIGITS team worked diligently, ensuring coordination across various aspects of the study, while also collaborating with healthcare providers, EHR programmers, the University of Washington, social workers, and the app vendor.

Similarly, the IMP^2^ART trial emphasised the importance of partnerships, particularly with OPC, which enabled the employment of large-scale integrated EHR data across the country. The trial team consisted of members from multiple UK academic institutions which enabled the trial to benefit from a wide range of expertise. Additionally, the trial included significant Patient and Public Involvement (PPI), in order to incorporate patient perspectives into the trial design and implementation (Table 2).

## Discussion

### Summary of findings

The two case studies had distinct models for using routine data, with the core difference being the types of routine data that were sourced and managed externally or internally. The two trials used routine data to characterise the practice population of eligible patients, support clinical and economic outcome evaluation, facilitate audit and feedback, and assist in intervention delivery. Common facilitators included sufficient IT and hardware capacity, relatively low cost, centralized regulatory approval for multi-site studies, and strong collaboration and partnerships. Common barriers included administrative complexity, redundant bureaucratic processes, and challenges with data sharing requirements. Data quality can act as either a facilitator or a barrier.

The DIGITS trial benefited from the use of in-house data warehouses within an integrated healthcare system, which ensured good data quality and enabled preliminary data analyses. In contrast, the IMP^2^ART trial, which recruited a large sample size across the nation, employed an external research database to extract and integrate data from various EHR systems. The context-specific challenges for the DIGITS trial included multiple IRB applications for various trial activities, while the IMP^2^ART trial faced issues with legacy EHR systems, changes in coding standards, and superstructure barriers such as site-specific approvals and practice-level data privacy concerns.

### Strengths and limitations

In this study, we employed a mix of methods, including interviews, documentation review, and feedback workshops, to achieve data triangulation and enhance the credibility of our findings. Although the limited number of cases restricts the generalisability of our results, the contrasting contexts of these cases are of value. Together they represent two very different approaches to EHR data governance and ownership: one where data is controlled by a single organisation and another with multiple, independent organisations. Other approaches will fall somewhere between these extremes, making these contrasting cases useful for illustrating a spectrum of real-world scenarios.

Another limitation is the potential for researcher bias in the interpretation of qualitative data. Despite the fact that we conducted an inter-coder reliability test to mitigate this, the subjective nature of qualitative analysis means that the findings are inevitably influenced by the researchers' perspectives. Additionally, this study focused on contextual factors and did not capture variations in participants' views based on roles or experience. While we included a broad range of informants, there were additional team members whose perspectives could have provided further insights. For instance, input from the study sponsor during the planning stages and perspectives from the data extraction team could have added valuable context. Including these voices in future research might help build a more comprehensive understanding of the topic.

Meanwhile, the dynamic nature of healthcare technology will affect the relevance of these findings over time. For example, while data quality remains a current challenge, advancements such as more widespread standardised coding practices or the use of AI to interpret clinical narratives might help reduce this barrier in the future. Finally, this study marks the first application of the Trial Efficiency Pentagon to guide an in-depth case study of implementation trials. While it demonstrates the utility of the framework in structuring data collection, analysis, and results reporting, its application remains exploratory. In applying the Trial Efficiency Pentagon, no themes were identified that fell outside the five framework domains, suggesting that the framework provided sufficient scope to capture key aspects of routine data use and trial efficiency in the two cases examined. However, the absence of prior case studies utilizing this framework means there are no established benchmarks or comparison points to draw upon. As a result, we approached this work as an opportunity to test and reflect on the framework’s relevance and adaptability in the context of implementation trials. Given the nascent stage of this framework, further work is needed to optimise its use across a broader range of trial contexts.

### Interpretation of findings in relation to previously published work

The commonalities identified across both trials resonate with findings from existing literature and may be broadly applicable across different contexts. In terms of facilitators, it is widely acknowledged that using routine data (EHR in particular) helps in the characterisation of the patient population, assists in intervention delivery, and supports outcome assessment [4, 16]. Routine data has also become a common data source for health economic evaluations in recent years [17–19].

Our findings are supported by Friedman et al. [20] that a robust data infrastructure, coupled with sufficient IT and hardware capacity, is a critical factor for the meaningful use of routine data. These technological resources not only facilitate the efficient processing and analysis of data but also contribute to reducing costs when compared to traditional trial-specific data collection methods [21, 22]. Centralised regulatory approval is expected to streamline the approval process and increase overall trial efficiency [23]. Additionally, collaborative stakeholder engagement, encompassing both internal team members and external partners, emerged as a universal facilitator. The collective efforts and shared expertise across and beyond the trial teams were instrumental in addressing issues as they arose.

Both trials also encountered common barriers that challenged the efficient use of routine data. Although appropriate regulatory governance is essential, redundant bureaucratic processes can complicate the trial operation [4, 24], with unnecessary administrative tasks adding to the workload for trial staff, detracting from the efficiency gains offered by routine data. Both trials discussed data sharing challenges, in slightly different scenarios. Data sharing is often required by research publishers to promote transparency, yet the legitimacy of sharing routine data has raised concerns. Routine data, unlike trial-specific data, are not collected for research purposes, and in some cases individual patient consent for data sharing was either unclear or absent [24]. These concerns highlight broader ethical considerations in the use of routine data. On the other hand, one trial faced challenges in sharing data between collaborative organisations due to the legal complexities. This issue is not isolated; it is recognised in other studies that data sharing is complicated and costly when it involves sensitive information, patient privacy, and the technical intricacies of data processing [25].

Data quality emerged as both a facilitator and a barrier across the trials, which echoes the findings from our previously published systematic review [4]. In both trials, missing data were considered minimal for outcome assessment; however, there were concerns regarding the potential coding variations, data granularity and availability for supporting implementation outcome assessment and full health economic evaluations. This secondary use of data highlights the challenge of ensuring high data quality in implementation trials, as data collected primarily for administrative or financial purposes may not always align with the requirements for accurate outcome assessment in research.

### Contextual differences and implications

The context-specific differences are worth noting as they provide insights on why certain strategies worked in one context but not in another.

The DIGITS trial is conducted within one region of Kaiser Permanente, which is one of the largest U.S. integrated healthcare delivery systems, serving 12.5 million members in eight states [26]. In 2004, KP launched a comprehensive EHR platform built on Epic Systems, known as KP HealthConnect [27]. It integrates patient information across all care settings, connects billing and ancillary systems, and offers members personal health records [26, 27]. Operating within this well-established data infrastructure, the DIGITS trial was able to employ an adaptive data processing approach, conducting preliminary data analyses to inform trial design. Nevertheless, it also faced unique challenges in navigating the internal IRB applications for different trial activities, highlighting the need for more harmonised IRB processes. The efficiency gains from the DIGITS trial highlight the importance of investing in robust data infrastructure for enhancing trial flexibility and responsiveness. This is also one of the key efforts for developing a learning health system for more efficient, data-driven decision making and improved patient outcomes [28].

The IMP^2^ART trial is conducted within the UK NHS, a universal healthcare system that covers over 98% of the population. The primary care EHRs are population-based routine data systems that capture comprehensive longitudinal patient information [29]. OPC is a not-for-profit social enterprise which extracts anonymised primary care data as a quality improvement service, which are also made available for research purposes in the OPC Research Database. The 16.6 million patient records, offer a resource for large-scale research across diverse populations [30]. Within this context, the IMP^2^ART trial was able to include a large, geographically diverse sample size across the country. However, the use of routine data via an external OPCRD database introduced data protection concerns. Despite having all required approvals regarding data privacy and appropriate use of personal information, a number of practices were advised by their Data Protection Officers that they should not participate. Two practices withdrew from the trial citing data concerns, one of which was worried that the routine data might be sold to commercial companies, a concern recently voiced in the press [31].

In addition, although OPC integrates data from all major UK GP software systems (EMIS, Vision and SystmOne), some legacy EHR systems were not able to be extracted as the information governance requirements for allowing individuals to ‘opt-out’ of data sharing were not compatible with new algorithms [32, 33]. Since 2018, primary care practices have been migrating to the SNOMED CT, gradually replacing the previous READ codes. While the new coding system allows greater interoperability, the accurate data mapping from historical READ codes has been a significant practical challenge [34].

The complexity of site-specific approvals is unique in the UK context, where NHS Health Boards and Trusts are responsible for overseeing and reviewing a trial's impact on local healthcare services, as well as approving the participation of general practices [35]. While they ensure that the general practices have the capability and resources to participate in the trials effectively and the trials are carried out in alignment with local healthcare needs [36], the local R&D approval process inevitably introduces multiple layers of bureaucracy. This is particularly evident in multi-site trials such as the IMP^2^ART trial, which had to navigate the diverse requirements of multiple Health Boards, each with its distinct procedures and criteria.

## Conclusion

Implementation trials are inherently population-based, population-driven, and population-serving. This study highlights how routine data has an impact on implementation trial efficiency across trial design, processes, superstructure, infrastructure, and stakeholder engagement. Embedding implementation trials within routine healthcare services maximises population reach. To improve trial efficiency in public healthcare systems, researchers must address technological and regulatory barriers to accessing data. In private healthcare systems, data use and access hinges on investing in robust IT infrastructure and ensuring comprehensive organisational commitment.

The distinct approaches in the US and UK reflect different healthcare systems, yet both countries have made significant strides in leveraging routine healthcare data to support evidence-based healthcare delivery and policy-making. By understanding the opportunities and limitations of using routine data in these two contexts, researchers can design and deliver future trials to optimise efficiency.

## Supplementary Information

Additional file 1: The consolidated criteria for reporting qualitative research (COREQ) checklist.

Additional file 2: Interview topic guide.

## Data Availability

The datasets generated and/or analysed during the current study are not publicly available due to restrictions of ethical approvals obtained for this study.

## Abbreviations

COREQ: Consolidated Criteria for Reporting Qualitative Research
EHRs: Electronic Health Records
HITECH: Health Information Technology for Economic and Clinical Health Act
IRB: Institutional Review Board
KP: Kaiser Permanente
NHS: National Health Service
OPC: Optimum Patient Care
PPI: Patient and Public Involvement
QMUL: Queen Mary University of London
SNOMED CT: Systematised Nomenclature of Medicine Clinical Terms
