# Supplementary material for: Impact of using routine healthcare data on the efficiency of implementation trials: a qualitative comparative case study
Source: Trials. 2026 Apr 11;27:373. doi: 10.1186/s13063-026-09706-3 (PMC13181941; doi:10.1186/s13063-026-09706-3)
Supplement: Supplementary file 1 — Additional file 1: The consolidated criteria for reporting qualitative research (COREQ) checklist. [file 13063_2026_9706_MOESM1_ESM.docx]

# Additional file 1: the consolidated criteria for reporting qualitative research (COREQ) checklist

Developed from: Tong A, Sainsbury P, Craig J. Consolidated criteria for reporting qualitative research (COREQ): a 32-item checklist for interviews and focus groups. Int J Qual Heal Care. 2018;19(6):349–357.

| **Topic** | **Item No** | **Guide Questions/Description** | **Page No.** |
| --- | --- | --- | --- |
| **Domain 1: Research team and reﬂexivity** | | | |
| *Personal characteristics* | | | |
| Interviewer/facilitator | 1 | Which author/s conducted the interview or focus group? | 7 |
| Credentials | 2 | What were the researcher’s credentials? E.g. PhD, MD | 7 |
| Occupation | 3 | What was their occupation at the time of the study? | 7 |
| Gender | 4 | Was the researcher male or female? | 7 |
| Experience and training | 5 | What experience or training did the researcher have? | 7 |
| *Relationship with participants* | | | |
| Relationship established | 6 | Was a relationship established prior to study commencement? | - |
| Participant knowledge of  the interviewer | 7 | What did the participants know about the researcher? e.g. personal  goals, reasons for doing the research | 7 |
| Interviewer characteristics | 8 | What characteristics were reported about the interviewer/facilitator?e.g. Bias, assumptions, reasons and interests in the research topic | 16-17 |
| **Domain 2: Study design** | | | |
| *Theoretical framework* | | | |
| Methodological orientation and Theory | 9 | What methodological orientation was stated to underpin the study? | 7 |
| *Participant selection* | | | |
| Sampling | 10 | How were participants selected? | 7 |
| Method of approach | 11 | How were participants approached? e.g. telephone, mail, Email | 7 |
| Sample size | 12 | How many participants were in the study? | 8 |
| Non-participation | 13 | How many people refused to participate or dropped out? Reasons? | - |
| *Setting* | | | |
| Setting of data collection | 14 | Where was the data collected? e.g. home, clinic, workplace | 7 |
| Presence of non-  participants | 15 | Was anyone else present besides the participants and researchers? | - |
| Description of sample | 16 | What are the important characteristics of the sample? e.g. demographic data, date | - |
| *Data collection* | | | |
| Interview guide | 17 | Were questions, prompts, guides provided by the authors? Was it pilot tested? | 8 |
| Repeat interviews | 18 | Were repeat interviews carried out? If yes, how many? | 8 |
| Audio/visual recording | 19 | Did the research use audio or visual recording to collect the data? | 7-8 |
| Field notes | 20 | Were ﬁeld notes made during and/or after the interview or focus group? | - |
| Duration | 21 | What was the duration of the interviews or focus group? | 7 |
| Data saturation | 22 | Was data saturation discussed? | 7-8 |
| Transcripts returned | 23 | Were transcripts returned to participants for comment and/or correction? | - |
| **Domain 3: analysis and ﬁndings** | | | |
| *Data analysis* | | | |
| Number of data coders | 24 | How many data coders coded the data? | 8-9 |
| Description of the coding  tree | 25 | Did authors provide a description of the coding tree? | 7-8 |
| Derivation of themes | 26 | Were themes identiﬁed in advance or derived from the data? | 7-8 |
| Software | 27 | What software, if applicable, was used to manage the data? | 8 |
| Participant checking | 28 | Did participants provide feedback on the ﬁndings? | 8 |
| Quotations presented | 29 | Were participant quotations presented to illustrate the themes/ﬁndings? Was each quotation identiﬁed? | Table 2 |
| Data and ﬁndings consistent | 30 | Was there consistency between the data presented and the ﬁndings? | 17 |
| Clarity of major themes | 31 | Were major themes clearly presented in the ﬁndings? | 10-16 |
| Clarity of minor themes | 32 | Is there a description of diverse cases or discussion of minor themes? | 10-16 |
