# Supplementary material for: Impact of using routine healthcare data on the efficiency of implementation trials: a qualitative comparative case study
Source: Trials. 2026 Apr 11;27:373. doi: 10.1186/s13063-026-09706-3 (PMC13181941; doi:10.1186/s13063-026-09706-3)
Supplement: Supplementary file 2 — Additional file 2: Interview topic guide. [file 13063_2026_9706_MOESM2_ESM.docx]

**Additional file 2: Interview topic guide**

# Section 1. Warm-Up Questions

1. Can you please introduce your role in the [xxx] trial? And how many years of experience do you have in conducting or being involved in healthcare trials?

2. What is your experience in using routine healthcare data?

**Section 2. Trial background**

- 1. **From your experience, did routine data have any impact on the selection of outcomes/endpoints?**
  2. **What (if any) impact does the use of routine healthcare data have on the selection of trial participants?** [only asked if RHD were used in participant identification]
  3. **How does the availability and quality of routine healthcare data influence the development of your statistical analysis plan for implementation trials?** (questions for statisticians)

- *How did you account for potential biases or missing data in routine healthcare data when devising your statistical analysis plan?*

*- Have there been any instances where the data significantly altered the initial statistical assumptions? If so, how were these situations addressed?*

*- How did the use of routine healthcare data impact your approach to determining sample size for implementation trials?*

*- Could you explain any adjustments or special considerations made in sample size calculations due to the characteristics of routine healthcare data?*

*-In your experience, has the availability of routine healthcare data allowed for more flexible or dynamic approaches to sample size estimation? How so?*

- 1. **Was the routine database provided by a third party or built in-house?** [infrastructure]
  2. **What human resources are available to support the use of routine data?/ What are the key stakeholders that contribute to using routine data (e.g. collection, analysis, interpretation etc.) in this trial?** [stakeholders]
  3. **Are there any policies or regulations that facilitate the use of routine data?** [superstructure]
  4. **Are there any policies or regulations that pose challenges to the use of routine data?**[superstructure]

**Section 3. Process of using routine data**

**3.1 Can you describe the process of requesting the routine data?** [superstructure]

*Prompts:*

- *Separate application form?*
- *Timeline?*
- *Detailed procedures*
  1. **How might applying for routine data affect the process of obtaining regulatory approvals?** [superstructure]
  2. **Was additional training needed to use routine data in this trial?** [trial process]
  3. **Was there any data linkage and how was it conducted?** [infrastructure]
  4. **Do you receive routine data at the end of the study or along the way? And can you describe the detailed procedures of data processing?** [infrastructure]
- *What are the initial steps taken once data are received?*
- *What processes does the routine data undergo once it is collected? For example, cleaning, validation, coding, etc.*
- *Can you describe the data flow from storage to analysis and report generation?*
- *Who processed the data?*
- *Are there any specific tools or software used to process routine data?*
- *Where is the routine data stored during the trial? Are there multiple storage locations or databases?*
- *How do you ensure the security and confidentiality of data in these storage systems?*
- *How was/is the data quality (accuracy/completeness)*
- *Is routine data shared with other stakeholders or systems during the trial?*
- *How routine data were/would be shared/archived at the end of the trial?*

**3.6 Are routine data used for participants' follow-up/retention? And what impact does it have on trial efficiency?**

**3.7. How much does it cost to use routine data? (for PI & Health Economists in particular)**

**-** Cost of data/cost of delivery of the trial

- What are the financial resources available to support the use of routine data?

**Section 4. Roles specific questions**

**4.1 As a xxx, what challenges do you face and what benefits do you gain when using routine data?**

**4.2 Based on your experience, what suggestions do you have to enhance the efficiency of implementation trials using routine data?**

**Closing**
